# Supplementary material for: Single platelet variability governs population sensitivity and initiates intrinsic heterotypic responses
Source: Commun Biol. 2020 Jun 4;3:281. doi: 10.1038/s42003-020-1002-5 (PMC7272428; doi:10.1038/s42003-020-1002-5)
Supplement: Supplementary file 1 — Supplementary Information [file 42003_2020_1002_MOESM1_ESM.pdf]

Supplementary Information

Single platelet variability governs population sensitivity and initiates intrinsic heterotypic responses

Supplementary Figures

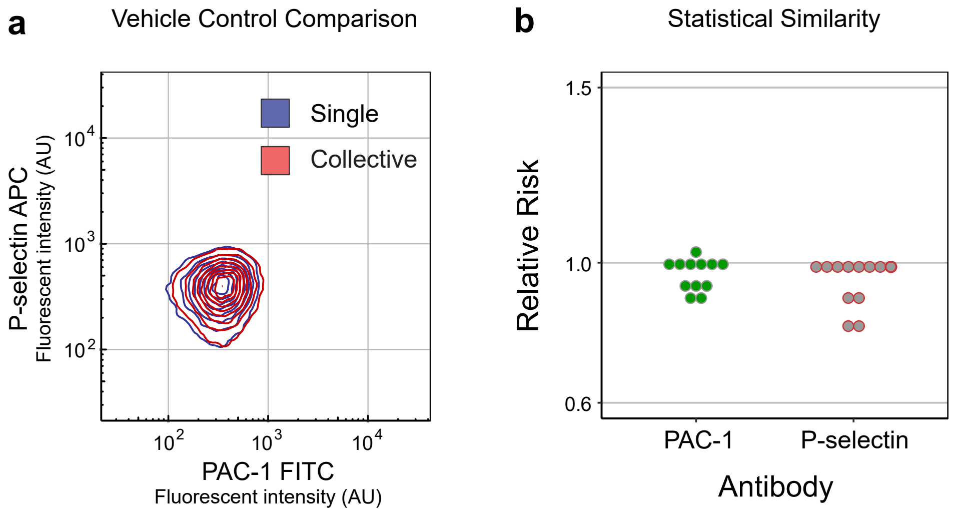

Supplementary Figure 1. *Vehicle control comparison*. The vehicle control signal from the droplet-confined platelets produces equivalent PAC-1 and P-selectin signal intensity distributions to platelet collectives (a). The relative risk scores comparing vehicle control single with collective platelet responses for the principle experiments involved in this study (b). A score of 1 indicates no difference. A relative risk below 1.0 indicate minor activation, highlighting the need for careful handling during the additional steps required for droplet encapsulation.

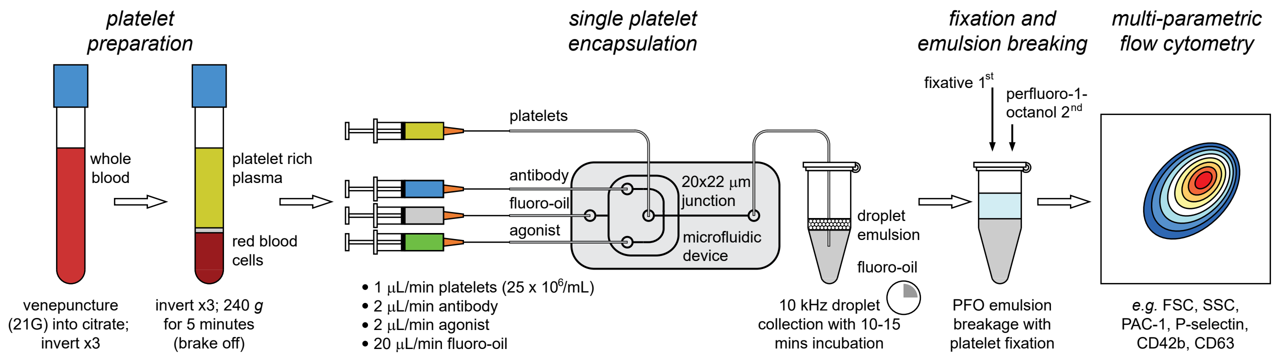

Supplementary Figure 2. *Illustrated protocol.* Venous blood draw through to platelet isolation, droplet encapsulation, incubation, platelet release by breaking the emulsion with fixation in readiness for flow cytometry. The analytical pipeline involves kHz droplet generation and kHz flow cytometry to deliver the throughput necessary to comprehensively interpret platelet population functional distributions.

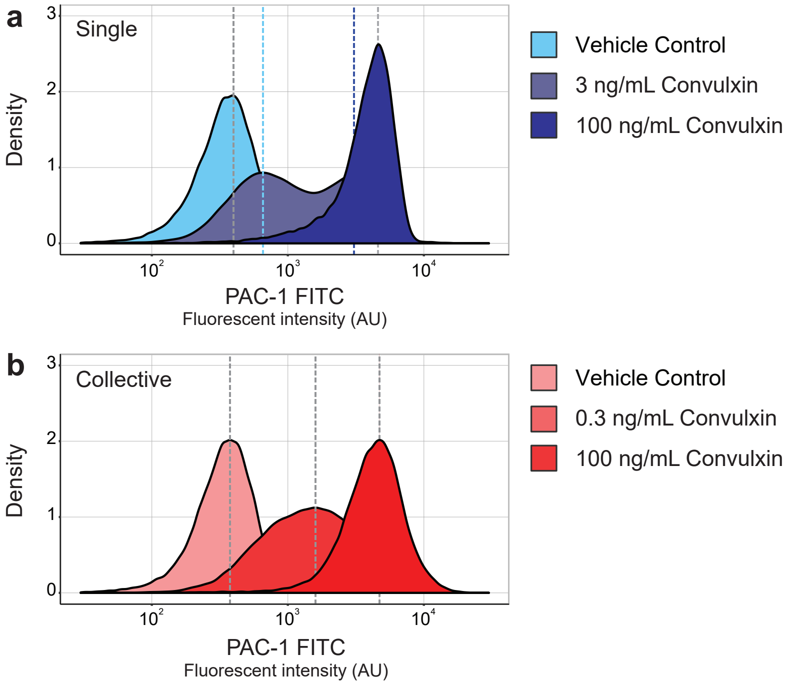

Supplementary Figure 3. *Transition states differ between single and collective platelet populations.* The peak maxima for inactive, transition and active states are identified with dashed lines for singular (a) and collective (b) platelet populations. The bimodal single platelet transition involves distinct inactive (light blue) and active (dark blue) signal maximas. The active signal maxima is higher than the maxima for collective platelets undergoing transition.

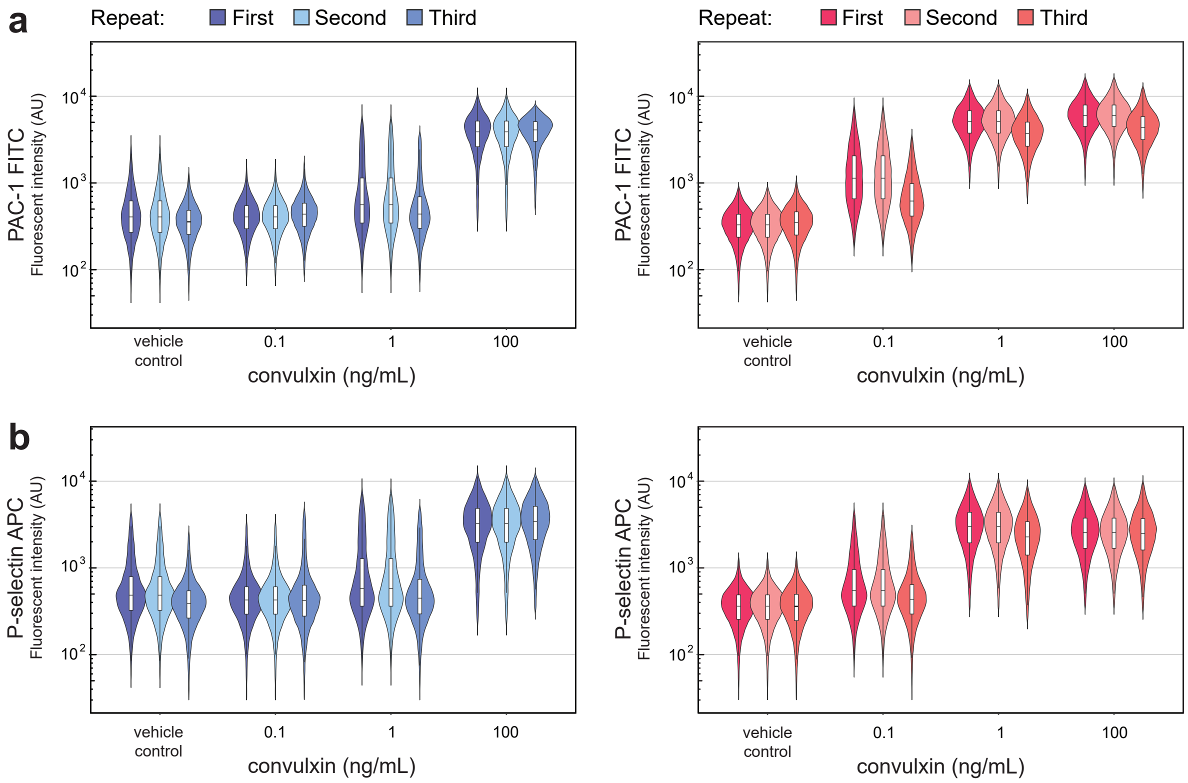

Supplementary Figure 4. *Donor reproducibility.* PAC-1 (a) and P-selectin (b) single (blues) and collective (reds) platelet responses to convulxin stimulation for the same donor measured three times over a 9 month period.

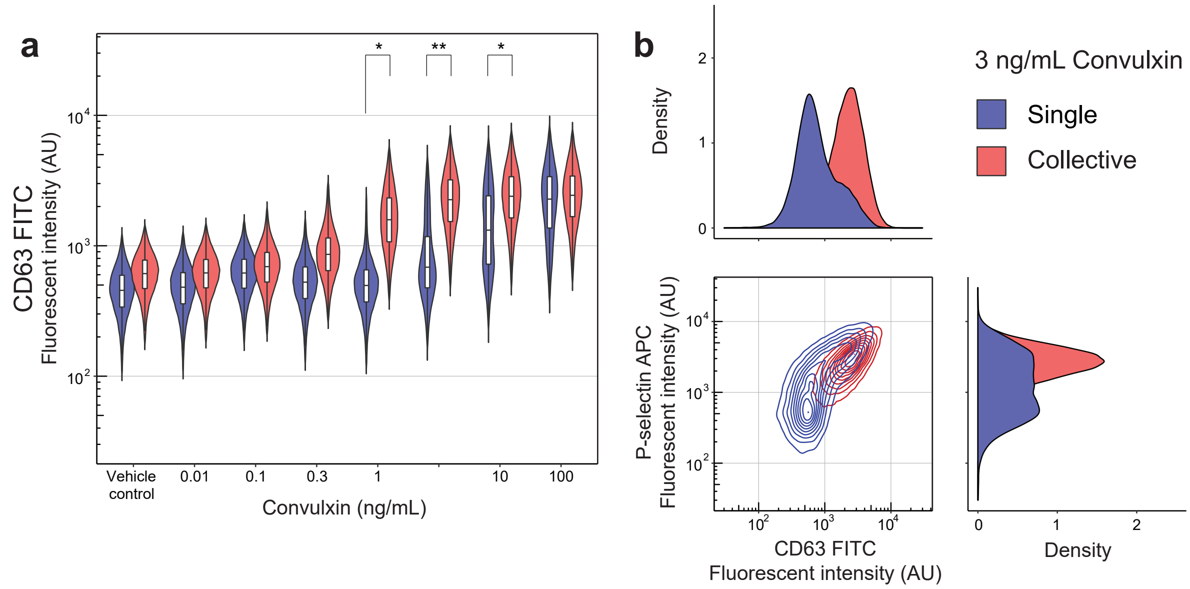

Supplementary Figure 5. *Alpha and dense granule secretions correlate.* Violin plots comparing the activation of single platelets with platelet collectives using a convulxin dose response experiment with the CD63 presentation, (dense granule secretion) end-point (a) (relative risk; *>2, **>5). Contour and density plots showing the correlation between alpha granule (P-selectin) and dense granule (CD63) secretion and the emergence of the hypersensitive single platelet subpopulation when stimulated with 3 ng/mL convulxin (b). The dense granule secretion pathway has a higher threshold for complete activation. For each single platelet condition, n=22,000–44,000 platelet events were measured, and n≈47,000 for the collective conditions.

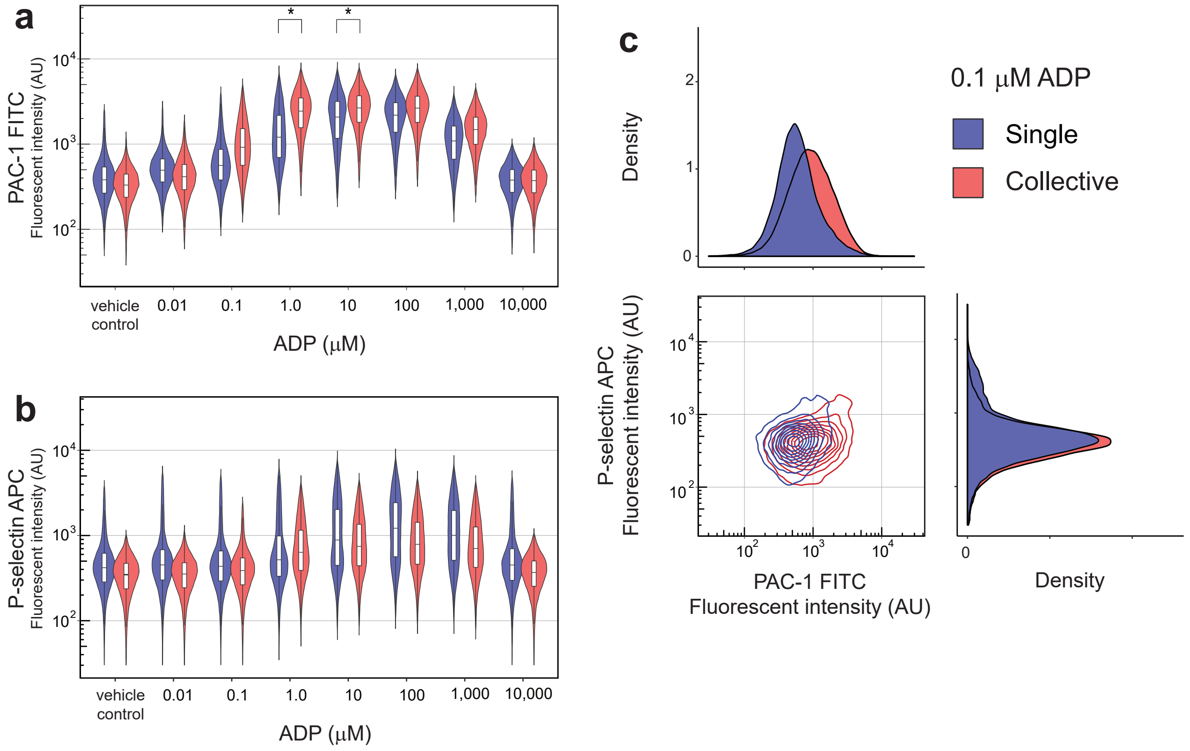

Supplementary Figure 6. *Minor variability in the response to ADP and minor collective sensitivity gains.* Violin plots comparing the activation of single platelets with platelet collectives using an ADP dose response experiment, with PAC-1 binding to activated $\alpha_{IIb}\beta_3$ (a) and P-selectin exposure (b) end-points (relative risk; *>2). Contour and density plots showing a small yet hypersensitive subpopulation for single platelets in droplets at 0.1 μM ADP (c). Desensitization with 1 and 10 mM ADP stimulations may result from rapid receptor internalization[1]. For each single platelet condition, n=16,000–43,000 platelet events were measured, and n≈49,000 for the collective conditions.

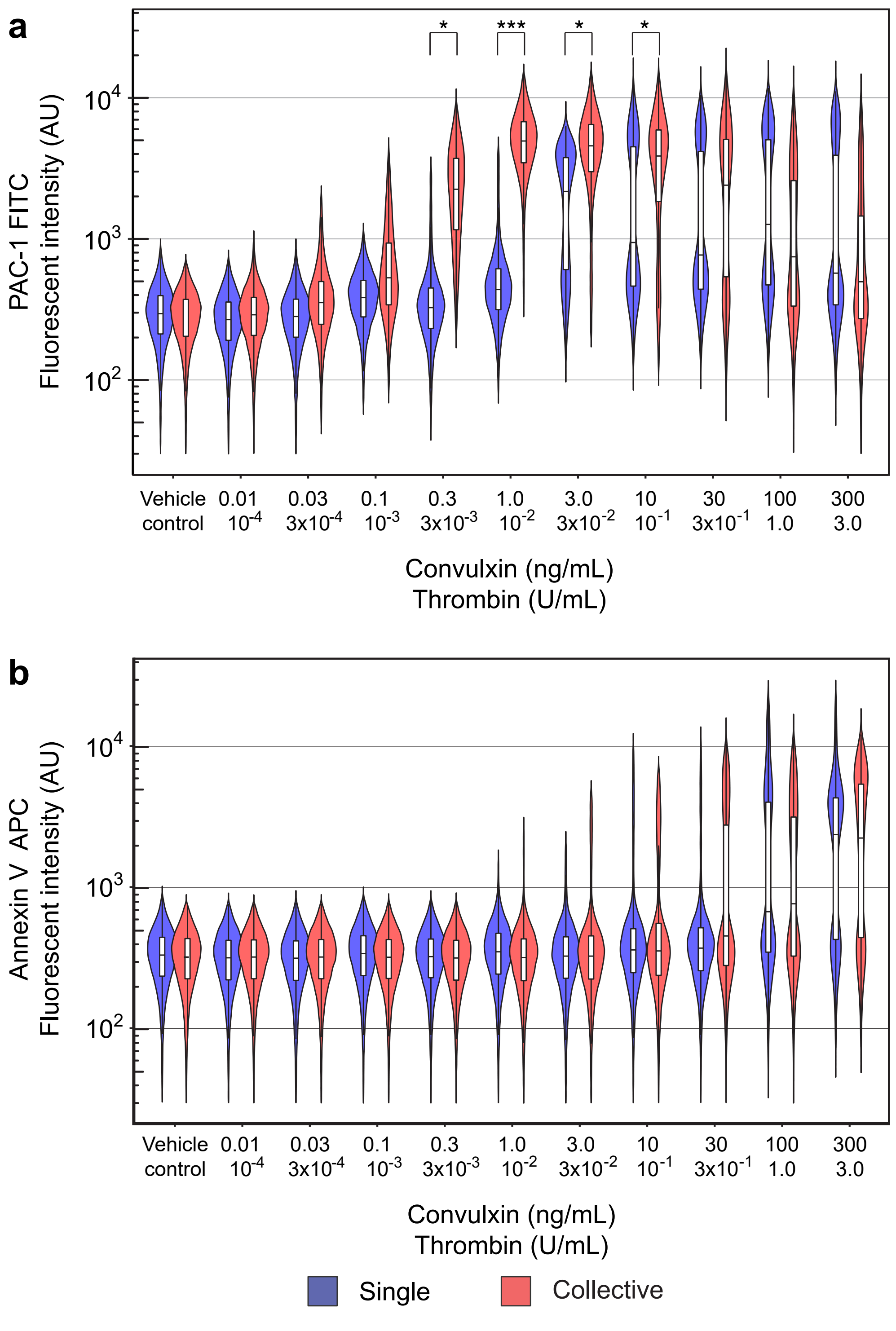

Supplementary Figure 7. *Dose responses for dual stimulated single and collective platelets.* PAC-1 binding (a) and P-selectin exposure (b) were used as end-points (relative risk; *>2, ***>10). For each single platelet condition, n=14,000–26,000 platelet events were measured, and n≈48,000 for the collective conditions.

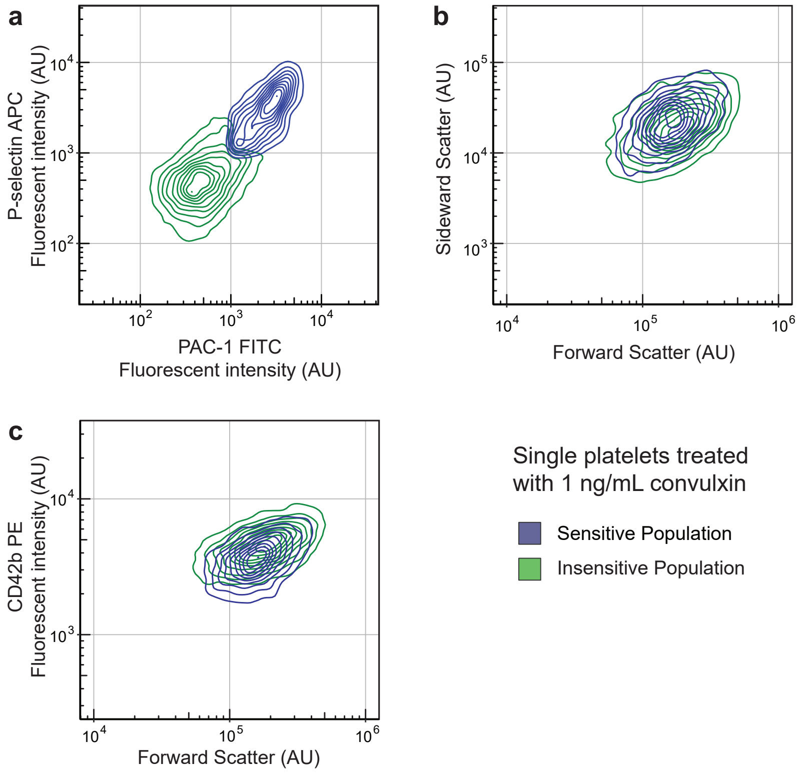

Supplementary Figure 8. *The nature of hypersensitivity requires further investigation.* Hypersensitive platelets ('sensitive population') identified by PAC-1 and P-selectin staining (a) cannot be distinguished by forward and side scatter properties from other ('insensitive') platelets (b). Platelet activation causes a minor CD42b signal reduction (c).

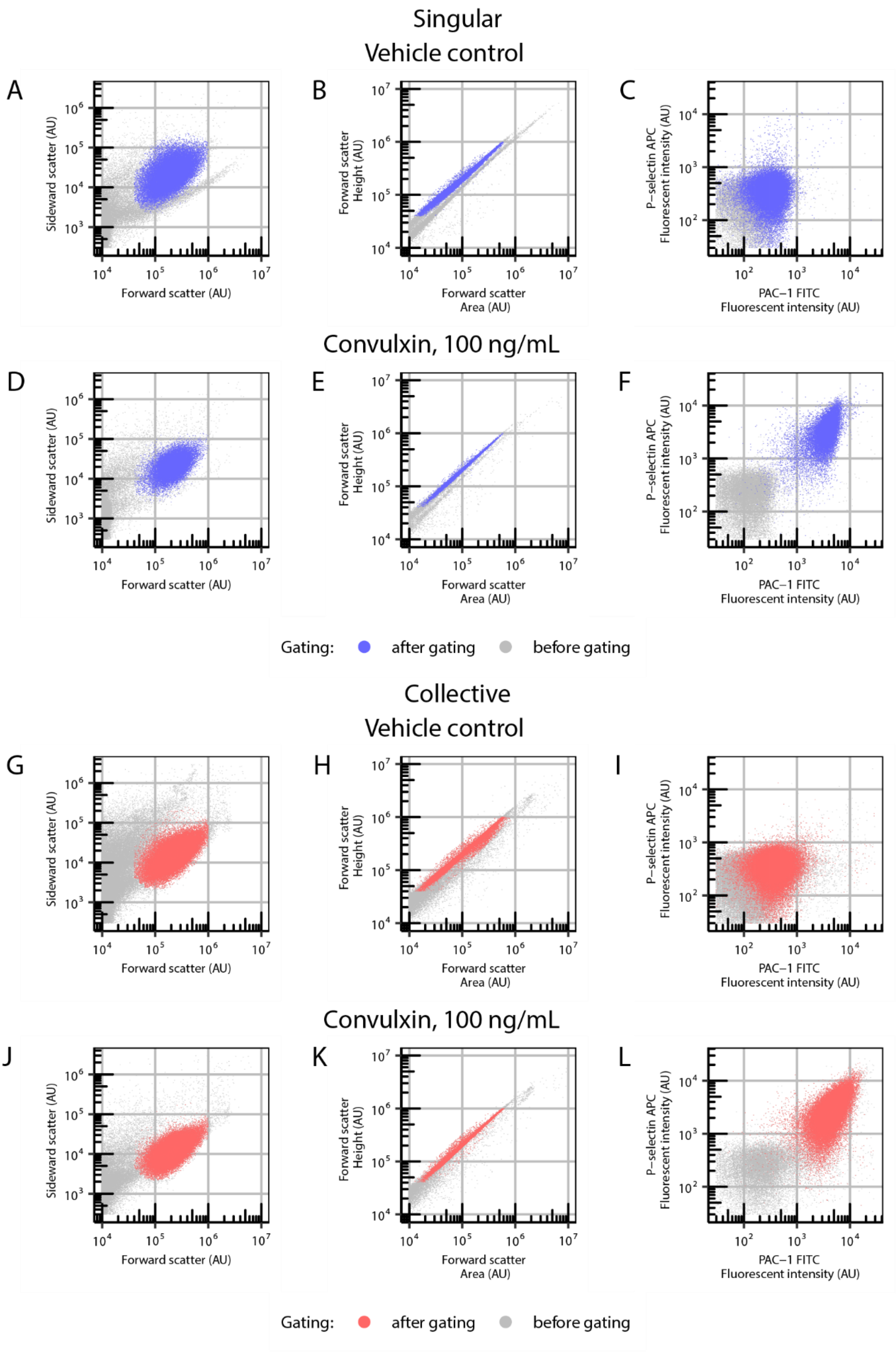

Supplementary Figure 9. *Gating strategy.* Single platelets identified from single (blue) and collective (red) experimental conditions compared with all measured events (grey). Vehicle control signals (A–C singular; G–I collective) are compared with platelets treated with 100 ng/mL convulxin (D–F singular; J–L collective).

Data Availability Statement

The raw cytometry .csv files are made available via FigShare (https://doi.org/10.6084/m9.figshare.12086103.v1).

REFERENCE

1 Baurand, A. *et al.* Desensitization of the Platelet Aggregation Response to ADP: Differential Down-regulation of the P2Y1 and P2cyc Receptors. *Thromb Haemost* **84**, 484-491, doi:10.1055/s-0037-1614049 (2000).
