# Supplementary material for: Single platelet variability governs population sensitivity and initiates intrinsic heterotypic responses
Source: Commun Biol. 2020 Jun 4;3:281. doi: 10.1038/s42003-020-1002-5 (PMC7272428; doi:10.1038/s42003-020-1002-5)
Supplement: Supplementary file 3 — Reporting Summary [file 42003_2020_1002_MOESM3_ESM.pdf]

nature research

Corresponding author(s): Jonathan West

Last updated by author(s): May 3, 2020

Reporting Summary

Nature Research wishes to improve the reproducibility of the work that we publish. This form provides structure for consistency and transparency in reporting. For further information on Nature Research policies, see Authors & Referees and the Editorial Policy Checklist.

Statistics

For all statistical analyses, confirm that the following items are present in the figure legend, table legend, main text, or Methods section.

n/a	Confirmed	
☐	☒	The exact sample size (*n*) for each experimental group/condition, given as a discrete number and unit of measurement
☐	☒	A statement on whether measurements were taken from distinct samples or whether the same sample was measured repeatedly
☐	☒	The statistical test(s) used AND whether they are one- or two-sided<br>*Only common tests should be described solely by name; describe more complex techniques in the Methods section.*
☒	☐	A description of all covariates tested
☐	☒	A description of any assumptions or corrections, such as tests of normality and adjustment for multiple comparisons
☐	☒	A full description of the statistical parameters including central tendency (e.g. means) or other basic estimates (e.g. regression coefficient) AND variation (e.g. standard deviation) or associated estimates of uncertainty (e.g. confidence intervals)
☒	☐	For null hypothesis testing, the test statistic (e.g. *F*, *t*, *r*) with confidence intervals, effect sizes, degrees of freedom and *P* value noted<br>*Give P values as exact values whenever suitable.*
☒	☐	For Bayesian analysis, information on the choice of priors and Markov chain Monte Carlo settings
☒	☐	For hierarchical and complex designs, identification of the appropriate level for tests and full reporting of outcomes
☒	☐	Estimates of effect sizes (e.g. Cohen's *d*, Pearson's *r*), indicating how they were calculated

Our web collection on statistics for biologists contains articles on many of the points above.

Software and code

Policy information about availability of computer code

Data collection	not applicable
Data analysis	Raw flow cytometry data (before gating) exported as CSV, gating (CD42b for platelet indicator, doublet discrimination FSC-A vs FSC-H, platelet size FSC-H vs SSC-H), Shapiro-Wilk in Graphpad Prism and Graphs (ggplot2) + relative risk (epitools) in R.

For manuscripts utilizing custom algorithms or software that are central to the research but not yet described in published literature, software must be made available to editors/reviewers. We strongly encourage code deposition in a community repository (e.g. GitHub). See the Nature Research guidelines for submitting code & software for further information.

Data

Policy information about availability of data

All manuscripts must include a data availability statement. This statement should provide the following information, where applicable:

- Accession codes, unique identifiers, or web links for publicly available datasets
- A list of figures that have associated raw data
- A description of any restrictions on data availability

The raw cytometry .csv files are made available via FigShare (doi: 10.6084/m9.figshare.12086103).

Field-specific reporting

Please select the one below that is the best fit for your research. If you are not sure, read the appropriate sections before making your selection.

☒ Life sciences ☐ Behavioural & social sciences ☐ Ecological, evolutionary & environmental sciences

Life sciences study design

All studies must disclose on these points even when the disclosure is negative.

Sample size	no sample size determined, research entailed assay development then fundamental investigation combining data from 8 healthy donors on multiple occasions.
Data exclusions	not applicable
Replication	Replication experiment described in Supplementary Figure 4
Randomization	not applicable
Blinding	not applicable

Reporting for specific materials, systems and methods

We require information from authors about some types of materials, experimental systems and methods used in many studies. Here, indicate whether each material, system or method listed is relevant to your study. If you are not sure if a list item applies to your research, read the appropriate section before selecting a response.

Materials & experimental systems

n/a	Involved in the study	
☐	☒	Antibodies
☒	☐	Eukaryotic cell lines
☒	☐	Palaeontology
☒	☐	Animals and other organisms
☐	☒	Human research participants
☒	☐	Clinical data

Methods

n/a	Involved in the study	
☒	☐	ChIP-seq
☐	☒	Flow cytometry
☒	☐	MRI-based neuroimaging

Antibodies

Antibodies used	FITC conjugated PAC-1 (PAC-1 clone), APC conjugated CD62P (P-selectin, AK-4 clone), FITC conjugated anti-CD63 (H5C6 clone), PE conjugated CD42b (HIP1 clone) and Annexin V were obtained from BD Biosciences.
Validation	Commercially available antibodies that are standard for platelet function studies

Human research participants

Policy information about studies involving human research participants

Population characteristics	healthy volunteers, not using any anti-platelet medication (24 h free of NSAIDS and 2 weeks of aspirin)
Recruitment	by advertisement across the University including the hospital
Ethics oversight	institute ethics committee (ERGO 5538) and South Central – Hampshire B (REC: 14/SC/0211)

Note that full information on the approval of the study protocol must also be provided in the manuscript.

Flow Cytometry

Plots

Confirm that:

☒ The axis labels state the marker and fluorochrome used (e.g. CD4-FITC).

☒ The axis scales are clearly visible. Include numbers along axes only for bottom left plot of group (a 'group' is an analysis of identical markers).

☒ All plots are contour plots with outliers or pseudocolor plots.

☒ A numerical value for number of cells or percentage (with statistics) is provided.

Methodology

Sample preparation	Standard venepucture and platelet preparation as described in the Materials and Methods section of the manuscript
Instrument	BD Accuri C6
Software	R and GraphPad Prism
Cell population abundance	not applicable
Gating strategy	Gating: CD42b for platelet indicator, doublet discrimination FSC-A vs FSC-H, platelet size FSC-H vs SSC-H

☒ Tick this box to confirm that a figure exemplifying the gating strategy is provided in the Supplementary Information.
