# Supplementary material for: Single platelet variability governs population sensitivity and initiates intrinsic heterotypic responses
Source: Commun Biol. 2020 Jun 4;3:281. doi: 10.1038/s42003-020-1002-5 (PMC7272428; doi:10.1038/s42003-020-1002-5)
Supplement: Supplementary file 5 — Description of Additional Supplementary Files [file 42003_2020_1002_MOESM5_ESM.pdf]

Description of Additional Supplementary Files

File Name: **Supplementary Data 1**
Description: CAD file for replicating the droplet microfluidic device.
